# Insights on the Mechanisms of the Protective Action of Naringenin, Naringin and Naringin Dihydrochalcone on Blood Cells in Terms of Their Potential Anti-Atherosclerotic Activity

**DOI:** 10.3390/molecules30030547

**Published:** 2025-01-25

**Authors:** Teresa Kaźmierczak, Sylwia Cyboran-Mikołajczyk, Natalia Trochanowska-Pauk, Tomasz Walski, Paulina Nowicka, Dorota Bonarska-Kujawa

**Affiliations:** 1 Department of Physics and Biophysics, Faculty of Biotechnology and Food Sciences, Wrocław University of Environmental and Life Sciences, Norwida 25 St., 50-375 Wrocław, Poland; 2 Department of Biomedical Engineering, Faculty of Fundamental Problems of Technology, Wrocław University of Science and Technology, 27 Wyb. Wyspiańskiego St., 50-370 Wrocław, Poland; 3 Department of Fruit, Vegetable and Plant Nutraceutical Technology, Faculty of Biotechnology and Food Sciences, Wrocław University of Environmental and Life Sciences, Chełmońskiego St. 37, 51-630 Wrocław, Poland

**Keywords:** erythrocytes, peripheral blood mononuclear cells, platelets, model lipid membrane, lipophilicity, antiradical, antioxidant, antiplatelet, spectroscopic methods, microscopy

## Abstract

Atherosclerosis is caused by injury to the blood arteries and progressive oxidative stress. Blood cells play an important role in its development; thus, their protection is important. Naringenin (N) is documented to possess a protective action against atherosclerosis, and we hypothesize that its derivatives, naringin (Nr) and naringin dihydrochalcone (Nd), with slightly different structures, possess similar or better activity. Therefore, this research aimed to find the mechanism of protective action of N, Nr and Nd in relation to erythrocytes, peripheral blood mononuclear cells (PBMCs) and platelets in terms of their potential anti-atherosclerotic effect. Moreover, their physicochemical properties and the interaction of flavonoids with liposomes were studied. All flavonoids protected erythrocytes from AAPH- and H_2_O_2_-induced oxidation to varying degrees. None of them had a destructive effect on erythrocyte membrane, and they did not impact the metabolic activity of PBMC and platelets. Nr and Nd inhibited collagen-induced platelet aggregation better in tested concentrations than N. Studied compounds did not induce liposome aggregation, but N and Nd changed their dipole potential. Obtained results show that Nd possesses slightly better activity than N and may have a better potential health effect on blood cells, which is very important in the design of anti-atherosclerotic therapeutics.

## 1. Introduction

Year by year the number of people with cardiovascular diseases (CVDs) increases. The World Health Organization alerts that the number of deaths around the world caused by CVDs has risen from 12.1 million in 1990 to 17.9 million in 2019 [1]. Data from Poland says that in 2021, around 35% of deaths were caused by circulatory diseases [2]. Atherosclerosis is a leading cause of CVDs. It is a chronic inflammatory state, caused by injury to the blood arteries and progressive oxidative stress [3]. Blood cells, including erythrocytes, peripheral blood mononuclear cells (PBMCs) and platelets, play a significant role in the development of atherosclerosis. Due to their function and circulation in the vessels, they are constantly exposed to many damaging factors, including free radicals generated by the imbalance of oxidative–reductive processes. In pathological states, their function is disturbed and the progression of the disease appears. Therefore, it is important to find the substances, which protect them against the damage. Moreover, the membrane of cells is the first and often the only barrier that separates the cell from the extracellular environment [4]. Thus, it is important to study the interaction of substances with the cell membrane.

Synthetic drugs supporting the treatment of atherosclerosis and circulatory failure have a number of side effects, including impairing the functioning of the immune system. Thus, research has focused on substances such as flavonoids, which come from natural plant sources and cause much less serious side effects.

Naringenin (N), 4′,5,7-trihydroxyflavanone, is a flavonoid derived from citrus fruits [5,6] and is documented to have protective effects on the cardiovascular system in vivo [7,8,9]. For instance, it alleviates elevated levels of lactate dehydrogenase (LDH) and oxidative stress markers in rats [7]. Moreover, it inhibits the production of pro-inflammatory factors in macrophages and blood [10]. Structurally, N consists of two benzene rings (A and B) connected by a chromane ring (C ring). It has three hydroxyl groups: one at position 4′ on the B ring and two at positions 5 and 7 on the A ring. One of the glycosylated derivatives of N is naringin (naringenin-7-O-neohesperidoside, Nr). Nr has a rhamnoside group attached to the C7 in the A ring of N. Nr is also a main component of citrus fruits [5,6,11] and has been documented to diminish the symptoms indicative of progressive oxidative stress and atherosclerosis [8,12,13,14,15,16]. Another derivative of N is naringin dihydrochalcone (phloretin-4′-O-neohesperidoside, Nd). Nd, besides having rhamnoside attached to the same carbon in A ring, has an open C ring with a hydroxyl group at the C2’ position. Nd is mostly known in the literature as a common sweetener [17]. However, some research suggests it may also have a protective effect against CVDs [18].

There are relatively few studies examining the interactions of N and its derivatives with blood cells in vitro, despite significant involvement of blood cells in the progression of atherosclerosis. Furthermore, derivatives of N, which have slightly modified structures, may exhibit similar or even enhanced protective activity compared to N itself. However, these derivatives have been less extensively studied in the context of their interactions with blood cells in vitro. Understanding the mechanisms of action of these substances, particularly their interactions with cells and cell membranes, as well as their structure–activity relationships (SARs), is crucial before considering them as therapeutic agents [19]. Therefore, the aim of this research was to investigate the interactions of naringenin, naringin and naringin dihydrochalcone with certain blood cells and model lipid membranes (liposomes). The results will provide insights into their potential mechanisms of action in protecting blood cells from damage that may contribute to atherosclerosis progression. Additionally, this study expands the understanding of how these interactions are related to the chemical structures of these compounds. The findings confirm that N derivatives, especially Nd, have better protective activity against blood cells than N.

## 2. Results

### 2.1. Determination of the Partition Coefficient Between 1-Octanol and Water (logP_o/w_) of Flavonoids and Their Solubility in Water

Determined partition coefficients between 1-octanol and water (logP_o/w_) for N, Nr and Nd are listed in Table 1. Obtained values were compared to the results derived from SwissADME tool [20] and MoleInspiration Cheminformatics. The predicted solubility in water was determined using SwissADME (Consensus logS).

The highest experimental partition coefficient (logP_o/w_) value was obtained for N, which is closely aligned with predictions from the SwissADME and MoleInspiration tools (Table 1). Conversely, the lowest logP_o/w_ was gained for Nr (Table 1). Although, the value predicted from the modelling tools showed a similar tendency to be low, they did not align as closely with the experimentally obtained value for Nr as they did for N. The logP_o/w_ value for Nd was intermediate between N and Nr (Table 1). However, the consensus logP was the smallest among all tested compounds. Interestingly, milogP for Nd was higher than for Nr, showing a similar pattern to the experimentally obtained results (Table 1).

The solubility of the flavonoids in water was determined based on the values of logS, obtained from the SwissADME tool. The highest consensus logS was obtained for Nr (−2.63) and the lowest for N (−3.63) (Table 1). LogS for Nd was slightly lower than for Nd (−2.53) (Table 1).

### 2.2. Antiradical Activity of Flavonoids

Table 1 presents the percentages of DPPH reduction by the flavonoids in the concentration of 100 μM. Besides flavonoids, standard antioxidants, L-(+)-ascorbic acid (AA) and butylated hydroxyanisole (BHA), reduced DPPH by 92.8 ± 0.5% and 87.4 ± 3.8%, respectively, in the same concentration. The results indicate that the tested flavonoids exhibit low antiradical activity (Table 1) compared to the standard antioxidants AA and BHA. DMSO, in which the tested compounds were dissolved, have no impact on DPPH activity (reduction by 0.7 ± 0.2%). Among the tested flavonoids, Nd demonstrated the highest antiradical activity (Table 1).

### 2.3. Interaction of Flavonoids with Human Erythrocytes

#### 2.3.1. Hemolytic Activity of Flavonoids and Their Impact on the Osmotic Resistance of the Erythrocytes

**Figure 1:**
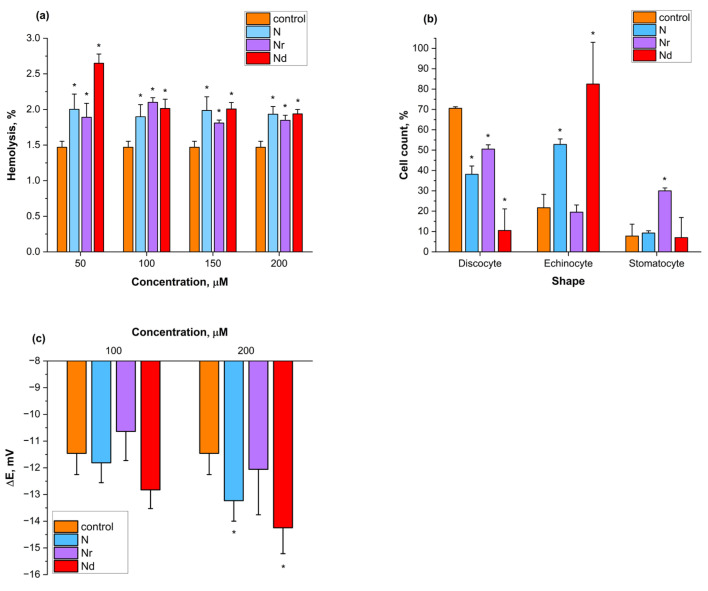
Effect of naringenin (N), naringin (Nr) and naringin dihydrochalcone (Nd) on different parameters of the erythrocytes: hemolytic activity of compounds (**a**), shapes induced with 100 μM of compounds (**b**); and trans-membrane potential (ΔE) (**c**). *—statistically significant differences between control and flavonoid-modified erythrocytes with *p* < 0.05.

N, Nr and Nd were not toxic to erythrocytes. Tested flavonoids did not induce significant hemolysis, as shown in Figure 1a. Furthermore, none of them affected the osmotic resistance of erythrocytes. The C_50_ values for the tested compounds were not statistically different from the control sample, in which the cells were treated with DMSO, and they were as follows: 0.47 ± 0.01% (control), 0.48 ± 0.004% (N), 0.48 ± 0.01% (Nr), and 0.46 ± 0.01 (Nd) in the concentration of 100 μM. The example of hemolytic curves for the control erythrocytes and Nd-treated are presented in Figure S1 (Supplementary Materials). 

#### 2.3.2. Changes in the Shape of the Erythrocytes Induced by Flavonoids

Figure 1b illustrates the percentage of blood cells exhibiting various shapes after treatment with the tested flavonoids in the concentration of 100 μM. N and Nd induced the formation of echinocytes, whereas Nr led to the formation of stomatocytes (Figure 1b).

#### 2.3.3. Effect of Flavonoids on the Trans-Membrane Potential of the Erythrocytes

The changes in trans-membrane potential (ΔE) of the erythrocytes, modified with N, Nr and Nd in the concentration of 100 and 200 μM are presented in Figure 1c. The results indicate that in the concentration of 200 μM only N and Nd reduced the trans-membrane potential compared to the control erythrocytes (Figure 1c). At the lower concentration, no statistically significant changes in trans-membrane potential were observed for erythrocytes modified with any of the tested flavonoids (Figure 1c).

#### 2.3.4. Inhibition of Free Radical-Induced Hemolysis by Flavonoids

**Figure 2:**
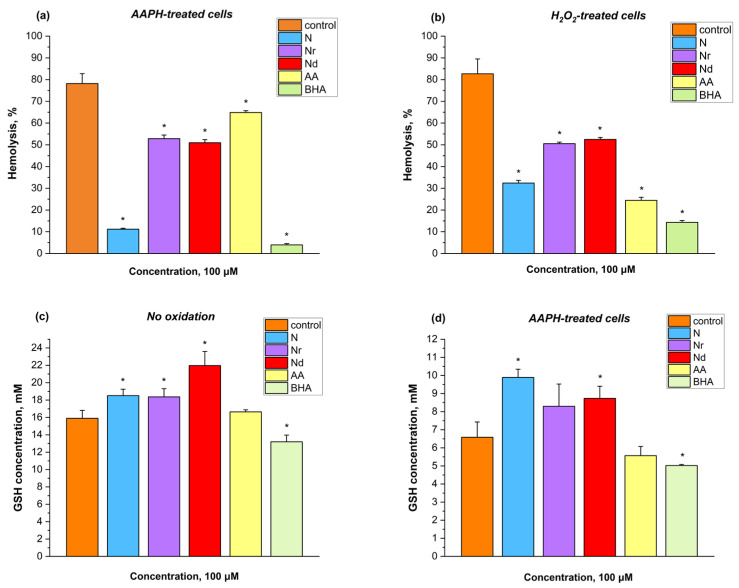
Effects of 100 μM of naringenin (N), naringin (Nr), naringin dihydrochalcone (Nd), L-(+)-ascorbic acid (AA), butylated hydroxyanisole (BHA) on the: percentage of AAPH-induced hemolysis (**a**), percentage of H_2_O_2_-induced hemolysis (**b**), glutathione (GSH) concentration (**c**) and GSH concentration after AAPH-treatment (**d**) in erythrocytes. *—statistically significant differences between control and compound-modified erythrocytes with *p* < 0.05.

The graphs presented in Figure 2 illustrate the percentage of hemolysis induced by 100 mM AAPH (Figure 2a) and 400 mM H_2_O_2_ (Figure 2b) after the modification of erythrocytes with flavonoids and standard antioxidants (AA and BHA) in the concentration of 100 μM. All tested flavonoids inhibited hemolysis induced by both free radical-inducers (Figure 2a,b). However, N demonstrated the highest activity, reducing AAPH- and H_2_O_2_-induced hemolysis by approx. 67% (Figure 2a) and 50% (Figure 2b), respectively. Nr and Nd exhibited similar levels of activity against both oxidants, though their effects were weaker than those obtained for N. They reduced AAPH-induced hemolysis by around 26% (Figure 2a) and H_2_O_2_-induced hemolysis by approximately 31% (Figure 2b). The results indicate that AA had significantly lower antioxidant activity compared to BHA (Figure 2a,b). Furthermore, AA was less effective than Nr and Nd against AAPH-induced hemolysis (Figure 2a). However, AA proved to be a better antioxidant than all tested flavonoids in protecting against H_2_O_2_-induced hemolysis (Figure 2b).

#### 2.3.5. Ability of Flavonoids to Prevent the Glutathione Oxidation in Human Erythrocytes

Figure 2 presents the millimolar concentration of reduced glutathione (GSH) in erythrocytes after treatment with N, Nr, Nd, AA and BHA (Figure 2c) and under AAPH-induced oxidative stress conditions (Figure 2d). All of the tested flavonoids increased the concentration of GSH in erythrocytes compared to the control cells, with Nd having the most pronounced effect (Figure 2c). No statistically significant changes were observed between the GSH concentration in the control erythrocytes and AA-treated erythrocytes. However, BHA significantly decreased the level of GSH in the erythrocytes (Figure 2c). In cells treated with AAPH, the inducer decreased the concentration of GSH almost in half compared to the non-treated erythrocytes (Figure 2d). However, this effect was not strong for the N- and Nd-treated cells, as it prevented its oxidation (Figure 2d). Nr did not alter the GSH concentration statistically, compared to the control erythrocytes (Figure 2d).

### 2.4. Effect of Flavonoids on the Mitochondrial Activity of the Human Peripheral Blood Mononuclear Cells (PBMCs) and Human Platelets

**Figure 3:**
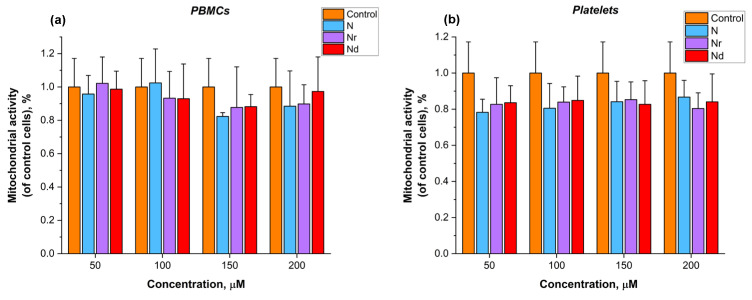
Mitochondrial activity (%) of human peripheral blood mononuclear cells (PBMCs) (**a**) and platelets (**b**) in relation to control cells after 24 h incubation with naringenin (N), naringin (Nr) and naringin dihydrochalcone (Nd) in different concentrations.

The effect of N, Nr and Nd on human peripheral blood mononuclear cells’ (PBMCs) and platelets’ mitochondrial activity in relation to control cells after 24 h of incubation is shown in Figure 3. The results indicate that none of the flavonoids significantly affected the mitochondrial activity of PBMCs (Figure 3a) or platelets (Figure 3b).

### 2.5. Interactions of Flavonoids with Human Platelets

#### 2.5.1. Ability of Flavonoids to Induce Apoptosis and Necrosis in Platelets

The ability of flavonoids to induce apoptosis and necrosis in platelets was assessed using flow cytometry. The results are presented in Table 2 as the percentages of apoptotic cells in the control sample (untreated cells) and flavonoid-treated samples. The representative results obtained from the flow cytometry measurements are presented in Figure S2 (Supplementary Materials). None of the flavonoids caused apoptosis or necrosis of the platelets in the concentration of 100 μM (Table 2).

#### 2.5.2. Antiplatelet Activity of Flavonoids

**Figure 4:**
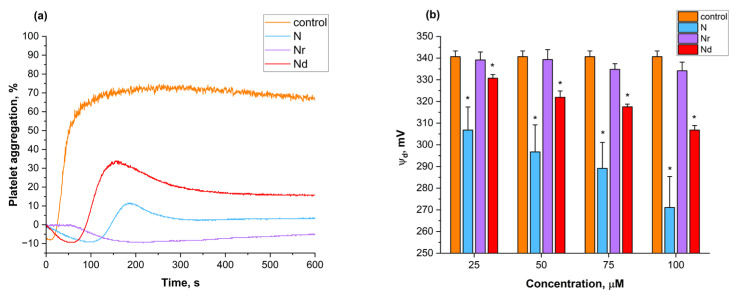
Aggregation curves for control platelets and platelets modified with 50 μM of naringenin (N), naringin (Nr) and naringin dihydrochalcone (Nd) (**a**). Changes in dipole potential (ψ_d_) of the liposomes, modified with increasing concentration of flavonoids (**b**). *—statistically significant differences between control and flavonoid-modified liposomes with *p* < 0.05.

The percentages of platelet aggregation of control and flavonoid-modified platelets in increasing concentrations used are shown in Table S1 (Supplementary Materials). The aggregation curves for N, Nr and Nd at a concentration of 50 μM are shown in Figure 4a. The aggregation was induced by the collagen. All tested flavonoids significantly inhibited the collagen-induced platelet aggregation. Nr demonstrated the highest antiplatelet activity, causing complete inhibition of aggregation at the lowest tested concentration (Table S1). Nd exhibited stronger antiplatelet activity than N, as it completely inhibited aggregation at the concentration of 75 μM compared to N at 100 μM (Table S1).

### 2.6. Interactions of Flavonoids with Model Lipid Membrane

#### 2.6.1. Effect of Flavonoids on the Hydrodynamic Diameter (HD) and Polydispersity Index (PDI) of the Liposomes

The results of the effects of N, Nr and Nd on the hydrodynamic diameter (HD) and polydispersity index (PDI) of liposomes prepared from lipids extracted from human erythrocyte membrane are shown in Table S2 (Supplementary Materials). No statistically significant differences were observed between HD and PDI values for liposomes modified with flavonoids in the concentration of 25 µM and 50 µM (Table S2).

#### 2.6.2. Effect of Flavonoids on Liposomes’ Dipole Potential (ψ_d_)

Changes in the dipole potential (ψ_d_) of liposomes modified with increasing concentrations of N, Nr and Nd are shown in Figure 4b. Only N and Nd significantly decreased the dipole potential, with N causing the greatest reduction in potential. The decrease in potential for N and Nd was concentration-dependent (Figure 4b).

## 3. Discussion

In recent years, nature-derived substances have gained increasing popularity in treating various diseases including atherosclerosis. The widespread availability of these substances and their lower toxicity than chemically synthesized compounds make them an attractive alternative to synthetic pharmaceuticals. As a result, the search for such substances continues to expand. This study aimed to investigate the mechanisms of action of the flavonoids N, Nr, and Nd on erythrocytes, peripheral blood mononuclear cells, and platelets, which may contribute to their protective effects against atherosclerosis. Additionally, the study was conducted on model lipid membranes (liposomes) composed of erythrocyte membrane lipids. This research also aimed to explore potential correlations between the chemical structure of these flavonoids and their biological activity on blood cells. It explored the potential molecular mechanisms of action of N, Nr and Nd.

The solubility of the compounds in the water was determined based on the SwissADME model tool, which calculates the logS value. The results showed that all of the flavonoids possessed the consensus logS values between −3.63 and −2.43 (Table 1). The values of logS between −4 and −2 are considered to characterize the water-soluble substance. Therefore, the solubility of the compounds in water and other water buffers is considered good [20].

The biological activity of a substance strictly correlates with its ability to interact with the cell membrane; therefore, its lipophilic character. The lipophilicity is assessed based on the ability of a substance to spontaneously diffuse between two chemically distinct phases: a nonpolar octanolic phase and a polar aqueous phase. Thus, the partition coefficient between 1-octanol and water (logP_o/w_) for the N, Nr and Nd was determined [21]. Positive values of the coefficient indicate a more lipophilic nature of the substance, suggesting a better interaction with the cell membrane and the potential passive diffusion through it. In contrast, negative values of the partition coefficient indicate a stronger interaction of the substance with the polar phase, often correlating with its presence in body fluids such as plasma. The logP_o/w_ values obtained experimentally were compared with those predicted by bioinformatics tools: SwissADME [20] and MoleInspiration Cheminformatics. Both tools provide preliminary results for studying potential therapeutic substances. The consensus logP value, derived from SwissADME, is the arithmetic mean of coefficients derived from several different mathematical models, predicting the lipophilic character of the substance. The MoleInspiration tool provides information about the partition coefficient (milogP). The prediction of the milogP value is based on group contribution, which takes into account possible intramolecular hydrogen bonds and charge interactions within the molecule. In this study, the highest partition coefficient value was obtained for N (Table 1). The consensus logP value and milogP are very similar to the values determined experimentally and reported in the literature [22]. In contrast, the lowest logP_o/w_ value was obtained for Nr (Table 1). Nd exhibits a higher partition coefficient than Nr but lower than N (Table 1). To date, no experimental logP values for Nr and Nd have been reported. However, both this study and results obtained by Rothwell et al. [22] have demonstrated that sugar moieties in the flavonoids, such as rhamnoside attached to Nr and Nd, have a greater impact on the partition coefficient than the number and position of hydroxyl groups. The rhamnoside has a much lower milogP value (−3.92) compared to the hydroxyl group (−0.27), as calculated using MoleInspiration, and corresponds to previously published data [22]. However, the rhamnoside in Nd has a smaller impact, as the open chromane ring increases the lipophilicity of the compound. This explains the higher logP values for Nd compared to Nr (Table 1). Based on the results, it can be concluded that Nr has a less lipophilic character than N and Nd and may interact with the cell membrane to a lesser extent. The differences between experimentally obtained and model-predicted values for Nr and Nd are attributed to various factors, including the potential for the compound to decompose in 1-octanol or differences in the approaches used in the logP prediction.

The ability of flavonoids to neutralize free radicals is crucial as it demonstrates their protective potential as antioxidants. This can be assessed in experiments measuring their capacity to reduce stable free radicals. In this study, N, Nr and Nd were tested for their ability to react with the stable radical 2,2-diphenyl-1-picrylhydrazyl (DPPH). Although DPPH is an artificial radical and differs from other reactive oxygen and nitrogen species, it was confirmed that the results positively correlate with the ones obtained in antioxidant measurements and inhibition of lipid peroxidation in the cell membrane [23]. The mechanism of the reaction of substances with DPPH remains elusive, as it combines both the hydrogen atom transfer (HAT) and single electron transfer (SET) mechanisms, described by Gulcin and Alwasel [24]. The obtained results showed that, out of all tested flavonoids, the highest antiradical activity exhibits Nd. The antiradical activity of the flavonoids is strictly related to their structure, as postulated by, above all, Bors’s team [25]. Bors and coworkers postulated three rules, which give rise to the scavenging activity of the flavonoids: (1) the presence of the catechol group in the B ring, (2) the 2,3-double bond in the C ring conjugated with the 4-oxo group, and (3) the presence of the catechol group at C3 and C4 in the A ring conjugated with the 4-oxo group [25,26]. It has been postulated that the resulting antioxidant radical is more stable, and there is a presence of electron delocalization and the ability to produce intramolecular hydrogen bonds in flavonoids [26]. Moreover, the number of -OH groups in the flavonoids has a big impact on their antiradical activity. In this research, N as a flavanone possesses the lowest antiradical activity out of all tested compounds. The structure of N, but also Nr and Nd, does not strictly correlate with the Bors criteria. Additionally, it was mentioned that hydroxyl groups at C5 and C7 in flavanone, which are present in N, do not participate in the antiradical activity [23]. Moreover, it was also reported by González and Nazareno that the rate of DPPH bleaching by N, but also Nr, is slow [27]. Nr, the glycosylated form of N, having rhamnoside attached to the C7, also has lower activity, and the results correspond to those obtained by Nakamura et al. [23] and González and Nazareno [27]. Bors proposed that the sugar moiety attached to C7 in flavonoids does not have an impact on their antiradical activity [26]. This argues with the conclusions proposed by different groups, in which the glycosylated flavonoids possess much lower activity than their corresponding aglycons [15,28]. Therefore, it may be concluded that rhamnoside in Nr favors the stability of the resulting flavonoid antiradical, raising its scavenging activity. Conversely, Nd possesses better, but still lower, antiradical activity than both N and Nr. Nakamura and coworkers proposed that the 2′-OH group in the open ring of dihydrochalcones, including that in the structure of Nd, is the significant structural factor, corresponding to their higher radical scavenging activity [23]. According to the Bors, Michel and Stettmaier proposals, N and Nr possess lower antiradical activity than the Nd because of the slow formation of flavonoid antiradicals [29]. It is also worth mentioning that DMSO can be a radical scavenger by itself, as was reported by other researchers [30]. However, the control group gave a scarce level of scavenging, which can be also attributed to the sample dilution. Therefore, the synergic effect of DMSO, as a flavonoids solvent, on the antiradical activity of the flavonoids and standard antioxidants can be excluded.

Next, the effect of the flavonoids was studied on human erythrocytes. Red blood cells are a great model to study the cytotoxicity of the substances and the interaction of the compounds with the cell membrane [31]. First, the hemolytic activity of the flavonoids was examined. In the series of concentrations from 50 to 200 μM, no hemolysis was observed (Figure 1a). Even though the percentage of hemolysis is a bit higher for the flavonoids compared to the control sample, it is still at physiological level. Therefore, N, Nr and Nd are not toxic to the erythrocytes. Red blood cells are constantly exposed to changes in the osmotic imbalance in the blood. Any alternations can cause them to become disrupted and hemolyzed, which may have a negative impact on their biological function as oxygen transporters in the body. Moreover, released hemoglobin can be toxic to other cells. Therefore, the osmotic resistance experiment was performed. The purpose of this experiment was to find if the flavonoids can change the osmotic fragility of the erythrocytes. Results show that in the concentration of 100 μM, none of the tested flavonoids change the osmotic fragility of the erythrocytes in relation to the control cells. The example of the hemolytic curves for the control erythrocytes and Nd-treated ones are presented in Figure S1 (Supplementary Materials). Therefore, obtained results indicate that these flavonoids do not act destructively on erythrocytes and do not have an impact on their osmotic resistance.

The cell membrane is the first barrier that separates the erythrocyte from the environment. Therefore, the interaction of the compounds with it is important. The erythrocyte membrane is prone to deformation but at the same time retains its shape [31]. The membrane is composed of lipids, membrane proteins (trans-membrane, surface) and a cytoskeleton, which is different from those observed in other cells. The most important protein in the cytoskeleton that provides the shape of the erythrocyte is the band-3 protein. This protein is responsible for the deformity of the erythrocyte shape [31,32]. Bessis proposed a distinction between blood cell shapes, as illustrated with his scale. Mostly, erythrocyte shapes are divided into discocytes, echinocytes and stomatocytes [33,34]. More lipophilic molecules lead to the formation of stomatocytes, besides hydrophilic ones produce echinocytes [35,36]. In this research, the shapes of erythrocytes modified with N, Nr and Nd were observed under an optical microscope. To this day, no naringenin- and naringin dihydrochalcone-induced erythrocyte shape change has been reported in the literature. It was found that N and Nd induce the formation of echinocytes, with Nd having a higher impact on this shape induction (Figure 1b). The results show that both of these compounds possess greater affinity to the outer layer of the erythrocyte membrane than with the inner. Nd, having sugar moiety in its structure and being less lipophilic than N (Table 1), interacts with the outer layer of the erythrocytes, forming echinocytes. However, N, being the highest lipophilic compound out of all tested flavonoids (Table 1), may interlace in its hydrophobic layer. Yet, obtained results indicate that it interacts with the outer layer of the erythrocyte membrane, inducing echinocyte formation (Figure 1b). This can be attributed to its interaction with other membrane components such as glycocalyx. In accordance with our results, N was reported to interact mainly with an outer layer of the membrane, as explained by the EPR results [37]. Surprisingly, Nr induced the stomatocyte formation (Figure 1b), which is in agreement with the study reported by [38]. This flavonoid, as a more hydrophilic compound (Table 1), is proposed to interact mainly with the outer membrane. Therefore, the stomatocyte formation indicates that it may interact with the glycocalyx. These results are also confirmed by Selvaraj et al. [38]’s research.

The shape of erythrocytes is correlated to their stability and permeability [39]. The erythrocyte membrane is composed of both lipids and proteins, out of which ion channels such as band 3 protein states the most important component [39]. The membrane potential is a physical property of the cell, which characterizes the ion distribution between the inner and outer layers of the membrane [40]. Trans-membrane potential for normal human erythrocytes was reported to be between −10 and −15 mV [39]. Any changes in the lipid distribution in the membrane cause changes in the ion pumps work and eventually, trans-membrane potential alternations. Therefore, it is important to study the effect of compounds on this physical parameter. The membrane potential mostly depends on the level of Cl^−^ ions; therefore, it can be measured by its release or with the help of fluorescent probes, which are sensitive to its changes [41]. 3,3′-Dipropylthiadicarbocyanine Iodide (DISC_3_(5)) is a potentiometric fluorescent probe, which was used in this research to measure the changes in trans-membrane potential after the modification of erythrocytes with N, Nr and Nd. Figure 1c shows the results: 100 μM concentration of flavonoids do not change the trans-membrane potential of erythrocytes significantly, but 200 μM of N and Nd decrease it in relation to the control erythrocytes. The results correspond to the effect observed for the changes of erythrocyte shapes, where N and Nd induce echinocyte formation (Figure 1b), interplaying their ability to interact mostly with the outer layer of the membrane. The slight diminution in the trans-membrane potential for N and Nr samples corresponds to the higher level of extracellular potassium ions. Therefore, those two flavonoids may prevent its permeability through the membrane by, for example, blockage of potassium channels [42,43]. Even though Nr did not show any changes in the trans-membrane potential, it was shown before to prevent the calcium efflux of erythrocytes, depending on the potassium channel. Therefore, it may prevent the cells from shrinkage and death [42]. Thus, it is another confirmation that N and Nd interact mostly with the outer layer of the erythrocyte membrane as well as ion channels, whose function depends on the erythrocyte shape.

Oxidative stress has several consequences at both the molecular and cellular levels. Therefore, it is crucial to identify substances that protect cells and inhibit the propagation of oxidation. It has been reported that N and Nr possess a variety of protective properties, including antioxidant activity [7,12,15,28]. However, there is limited information about their protective effects on blood cells, particularly erythrocytes, which are constantly exposed to oxidative stress due to their role as oxygen transporters. Consequently, the potential of these compounds to inhibit oxidant-induced hemolysis was investigated. In the experiment, two inducers of free radicals were used: 2,2′-Azobis(2-methylpropionamidine) dihydrochloride (AAPH) and hydrogen peroxide (H_2_O_2_). AAPH generates free radicals at 37 °C, which causes lipid peroxidation [44]. In addition, H_2_O_2_ is an active radical itself and can also permeate through cell membranes [45]. Thus, it may exert harsher effects than AAPH. The results showed that N exhibited the highest antioxidant activity against both AAPH- (Figure 2a) and H_2_O_2_-induced (Figure 2b) hemolysis. The antiradical effect of antioxidants, including flavonoids, is attributed to their ability to stabilize free radicals, thereby halting the propagation of oxidation reactions [45]. N demonstrated greater antioxidant activity against AAPH radicals than against H_2_O_2_ (Figure 2), consistent with previous findings that N has better scavenging activity towards peroxyl radicals than hydroxyl radicals [28]. These results, however, do not align with the DPPH experiment, where N showed the lowest scavenging activity (Table 1). This discrepancy likely arises because cellular oxidation is a complex process involving multiple mechanisms. For instance, N may protect erythrocytes through various pathways. Studies have shown that this flavonoid can inhibit ferroptosis in BEAS-2B cells [46], a process dependent on reactive oxygen species and intracellular iron levels [47]. Since erythrocytes contain heme proteins, they may undergo ferroptosis during oxidation, and N could protect them from this process. Additionally, N may exhibit metal chelation or metal ion reduction activity, thereby preventing the Fenton reaction [28]. Both Nr and Nd displayed similar antioxidant activity against AAPH- (Figure 2a) and H_2_O_2_-induced (Figure 2b) hemolysis, though their effects were significantly weaker compared to N. This reduced activity may be due to the inability of Nr and Nd to penetrate cell membranes and neutralize intracellular H_2_O_2_, resulting in their interaction primarily with the outer cell layer. Their localization at the cell surface, likely due to their greater hydrophilicity relative to N, limits their interaction with radicals within the cellular environment, thus reducing their protective efficacy. The lower antioxidant effects of Nr and Nd may also be attributed to their sugar moieties, which could create structural hindrance, limiting access to radicals or preventing effective protection of cellular components from oxidation [28,48]. This observation is consistent with the DPPH results, where both compounds exhibited low antiradical activity compared to standard antioxidants (Table 1). Furthermore, studies have shown that Nr can inhibit methemoglobin production and prevent protein and lipid oxidation induced by Fe^3+^ [13,49,50]. Currently, there is no information available regarding the antioxidant properties of Nd on erythrocytes.

Glutathione (GSH) is a tripeptide that acts as a low molecular weight intracellular antioxidant. Its thiol group is a reactive moiety that interacts with radicals, and the reaction rates between glutathione and various radicals are notably high [51]. The reduced form of glutathione (GSH) is not harmful to cells; however, its oxidized form, glutathione disulfide, is harmful to cell components [52,53,54]. To gain further insights into the potential antioxidant mechanisms of flavonoids, an experiment was conducted to measure reduced GSH levels in erythrocytes. Initially, it was assessed whether flavonoids at a concentration of 100 μM could independently influence GSH levels in erythrocytes. Remarkably, all tested flavonoids increased the intracellular levels of reduced glutathione compared to the control (Figure 2c). The most significant effect was observed in cells treated with Nd, where the GSH concentration was the highest. In AAPH-treated samples, GSH concentration decreased almost by half (Figure 2d). However, this effect was not observable in the erythrocytes treated before with N and Nd. No differences were observed for Nr (Figure 2d). These findings align with the results from the DPPH assay, in which Nd exhibited the highest antiradical activity (Table 1). This suggests that flavonoids may protect erythrocytes by promoting glutathione reduction. Consequently, they are also capable of safeguarding cellular proteins that contain thiol groups in their structure [52,53,54]. The results for N are consistent with those previously reported by Harisa [55], who demonstrated that N increased the GSH/GSSG ratio in erythrocytes compared to control samples treated with the hemolytic agent paclitaxel. Furthermore, it has been found that flavonoids with a phenolic B ring, such as N, Nr, and Nd, oxidize GSH in vitro in the presence of peroxidases [56,57]. In conclusion, N, Nr, and Nd exert a stronger influence on GSH levels in erythrocytes compared to standard antioxidants. Thus, their antioxidant mechanism involves the prevention of glutathione oxidation, contributing to their protective effects against oxidative stress.

In addition to erythrocytes, peripheral blood mononuclear cells (PBMCs) and platelets play a significant role in the progression of many cardiovascular diseases. Toxicity studies of compounds, including flavonoids, should, therefore, also be conducted on these cell types. Although N and its derivatives have shown protective effects on erythrocytes, the concentrations used may pose a risk of toxicity to other cells [58]. Metabolic function is a key parameter in assessing cell viability. Accordingly, the effect of flavonoids on PBMCs and platelets was evaluated using the tetrazolium chloride (XTT) assay. The results, presented in Figure 3, indicate that none of the tested flavonoids reduced mitochondrial activity in PBMCs (Figure 3a) or platelets (Figure 3b) across increasing concentrations after 24 h of incubation. This finding aligns with previous research by Kocyigit and colleagues, who reported that only very high concentrations of naringenin (over 1200 μM) exhibit cytotoxic effects on PBMCs [58]. Similarly, Torcasio et al. demonstrated that Nr shows no toxicity toward peripheral blood mononuclear cells [59]. Therefore, N and its derivatives do not exhibit toxicity toward PBMCs or platelets, suggesting that they can be used safely at the concentrations studied.

The potential effects of N, Nr, and Nd on the induction of apoptosis and necrosis in platelets were also investigated using flow cytometry. Two fluorescent probes were employed in this experiment: Annexin FITC and propidium iodide (PI). Annexin FITC detects early-stage apoptosis in cells [60], while propidium iodide identifies cells in the necrotic stage [60,61]. The results presented in Table 2 indicate that none of the tested flavonoids induced apoptosis in platelets at a concentration of 100 μM. Platelet apoptosis negatively affects their aggregation ability. During platelet aggregation, phosphatidylserine from the inner layer of the platelet membrane is translocated to the outer layer, serving as a marker of apoptosis [62,63]. Thus, it can be concluded that all the tested flavonoids are safe for use, as they do not exhibit a procoagulant effect on platelets. These findings are consistent with other studies, which have confirmed that N and Nr do not induce platelet apoptosis [14,63].

In pathological conditions such as atherosclerosis, excessive platelet activity and the formation of the so-called atherosclerotic plaque can lead to clot building and, ultimately, clot detachment. In turn, such a clot may contribute to the formation of an infarction [3,64]. Hence, it is important during the atherosclerosis development and progression to act on platelets in a way that inhibits them from aggregation. The mechanisms of platelet aggregation activation are different and are based on both extracellular inductors, such as collagen or thrombin or inner, i.e., intracellular calcium concentration, phosphorylation cascades [64,65]. In this research, the activity of the flavonoids on collagen-induced platelet activation was studied. Collagen is a main inducer (agonist) of platelet activation, by binding with GP receptors on the platelet membrane surface [65]. The results of platelet aggregation (%) treated with increasing concentrations of flavonoids are shown in Table S1 (Supplementary Materials). Nr inhibited collagen-induced platelet aggregation almost completely in the concentrations used (Table S1, Supplementary Materials). N and Nd possess dose-dependent antiplatelet activity; however, a better effect in lower concentrations is observed by Nd. Complete inhibition of aggregation is observed for Nd at a concentration of 75 μM and for N at 100 μM (Table S1). The aggregation curves for N, Nr and Nd in the concentration of 50 μM are presented in Figure 4a. Flavonoids possess a different effect on the platelet aggregation inhibition, which can also correspond to their structure. Xiao and coworkers found that Nr inhibited aggregation of rabbit platelets in a dose-dependent manner [14], which is attributed to the release of intracellular calcium and inhibits the cascades leading to aggregation. However, the mechanism of antiplatelet action of the flavonoids may include both the occupation of the GPII/IIIa receptor by those compounds [66] and their binding with the agonist (collagen) with high affinity [67]. It was also proposed that the antiplatelet activity of flavonoids corresponds with their structure and also antiradical activity [67]. Our results show that the derivatives of N, containing sugar moieties, possess better antiplatelet activity than aglycone (Table S1). Furthermore, as the DPPH results showed, they also exhibit higher antiradical activity (Table 1). Apigenin, structurally similar to N, was found to bind to the collagen with high affinity, which explains its high antiplatelet activity [68]. Because of the similarity in structure between apigenin and naringenin, the mechanism of antiplatelet activity of the studied compounds may also be due to their interaction with the collagen itself and/or by interaction with collagen receptors on the platelet surface. Hence, the exact determination of the mechanism of action of N, Nr and Nd requires additional studies.

To find the possible mechanism of interaction of the flavonoids with the membrane lipid components, research with the usage of liposomes, composed of lipids extracted from the erythrocyte membrane, was performed. The effect of flavonoids on the hydrodynamic diameter (HD) and polydispersity index (PDI) of liposomes and changes in dipole potential (ψ_d_) were tested. The experiment showed that the tested flavonoids do not change the HD and PDI of the liposomes (Table S2, Supplementary Materials) in the concentration of 25 and 50 μM. This suggests that these flavonoids do not aggregate liposomes. It was mentioned by Danaei et al. that the monodisperse population of liposomes is described as PDI between 0.05 and 0.7 [69]. Therefore, based on the obtained PDI values ranging around 0.2, it can be concluded that the population of liposomes is monodisperse. The dipole potential is characterized by the dipole orientation of lipid polar groups and water molecules [70]. Compounds, including flavonoids, with a large dipole moment also attributed to its changes [71,72]. The results of the experiment in Figure 4b show that only N and Nd have an impact on the ψ_d_ of tested liposomes, decreasing its value in a concentration-dependent manner. Nr does not significantly change the dipole potential in relation to the control (Figure 4b). The results can be explained by the ability of the compounds to interact with the lipid bilayer and, therefore, their lipophilicity. N, as the most lipophilic-tested flavonoid (Table 1) may influence water absorption by the membrane, thus decreasing the ψ_d_ values. Nd, as a dihydrochalcone, has slightly lower lipophilicity than N (Table 1), and it decreases the dipole potential but with a smaller effect. Similar values were obtained for phloretin, a dihydrochalcone, which has also lipophilic character and induces the drop in ψ_d_ [71]. The results obtained for both flavonoids correspond to their decreasing effect on trans-membrane potential (Figure 1c) and changes in the shapes of erythrocytes (Figure 1b). Thus, they may change the water molecule distribution on the membrane surface. Other authors also have found that N and other dihydrochalcones decrease the dipole potential [72,73]. N, having only a slight effect on the shapes of erythrocytes (Figure 1b), does not change ψ_d_ (Figure 4b) and the trans-membrane potential (Figure 2c). Its lipophilicity is very low; therefore, it interacts with the membrane mostly on its surface.

## 4. Materials and Methods

### 4.1. Materials

#### 4.1.1. Compounds and Chemical Reagents

Flavonoids: (+/−)N, Nr and Nd were obtained from Merck (Dramstadt, Germany). Chemicals used for the preparations of the buffers are as follows: NaCl, KH_2_PO_4_ (Avantor Performance Materials, Gliwice, Poland), Na_2_HPO_4_·12H_2_O, NaH_2_PO_4_·H_2_O, sodium-EDTA, Tris (Chempur, Piekary Śląskie, Poland), KCl, NaOH (“STANLAB” SP. Z O.O., Lublin, Poland), NH_4_Cl, NaHCO_3_ (EUROCHEM BGD Sp. z o.o., Tarnów, Poland). 2,2′-Azobis(2-methylpropionamidine) dihydrochloride (AAPH), hydrogen peroxide (H_2_O_2_), 2,2-diphenyl-1-picrylhydrazyl (DPPH), L-(+)-ascorbic acid (AA), butylated hydroxyanisole (BHA), 5,5′-dithio-bis-(2-nitrobenzoic acid) (DTNB), glutaraldehyde, dimethyl sulfoxide (DMSO), immersion oil, 1-propanol, Lymphosep, RPMI-1640, Hank’s Balanced Salt Solution, phosphate buffer saline, 3,3′-Dipropylthiadicarbocyanine Iodide (DiSC_3_(5) and 4-[4-[(1E,3E)-4-[4-(dipentylamino)phenyl]buta-1,3-dienyl]pyridin-1-ium-1-yl]butane-1-sulfonate (Rh421) were purchased from Merck (Dramstadt, Germany). CaCl_2_ was purchased from Chempur, Piekary Śląskie, Poland. Methanol and ethanol were purchased from “STANLAB” SP. Z O.O. (Lublin, Poland). Trichloroacetic acid (TCA) was bought in Ubichem Plc (Eastleigh Hampshire, UK). Collagen was obtained from Chrono-Log (Havertown, PA, USA). Tetrazolium chloride (XTT), phenazine methasulfate (PMS), fetal bovine serum (FBS), Pen-Strep and Annexin V-FITC/PI apoptosis kit were obtained from ThermoFisher Scientific (Waltham, MA, USA).

#### 4.1.2. Biological Material

Human erythrocytes, peripheral blood mononuclear cells (PBMCs) and platelets were derived from the blood bank based on the scientific agreement between Wrocław University of Environmental and Life Sciences and Tadeusz Dorobisz Regional Centre for Blood Donation and Hemotherapy in Wrocław. Erythrocytes were obtained in a form of red blood cell concentrate or after isolation from the whole blood. PBMCs were obtained after isolation from the leukocyte-platelet coat, described in Section 4.2.3. Platelets were received in a form of platelet concentrate.

### 4.2. Methods

#### 4.2.1. Determination of the Partition Coefficient Between 1-Octanol and Water (logP_o/w_) of Flavonoids

The partition coefficients between 1-octanol and water (logP_o/w_) for N, Nr and Nd were determined according to the procedure described by Baluja, Kulshrestha and Movalia [21], after minor modifications. Compounds were dissolved in 1-octanol in the concentration of 100 μM, which was further used for dilution. For the determination of their logP_o/w_, 1 mL of 50 μM flavonoids in 1-octanol was shaken with 1 mL of distilled water for 3 h, at 1400 rpm, at room temperature. After two phases were separated, the absorption spectra of the compounds in 1-octanol were retrieved using a Specord 40 spectrophotometer (Analytik Jena Gmb, Jena, Germany). The partition coefficients were calculated based on the previously prepared standard curves.

#### 4.2.2. Antiradical Activity of Flavonoids

The determination of the antiradical activity of the flavonoids was performed based on their ability to bleach a stable radical—2,2-diphenyl-1-picrylhydrazyl (DPPH), measured according to the procedure described before by Goupy et al. [74], after minor modifications. The experiment was performed in a 96-well plate for 200 μL. As antioxidant standards, AA and BHA dissolved in DMSO were used. The plate was shaken for 30 min, at 400 rpm, at room temperature in darkness. The absorbance was measured on the Agilent BioTek Epoch Microplate Spectrophotometer (Agilent Technologies, Santa Clara, CA, USA) at 517 nm.

#### 4.2.3. Cell Isolation

Erythrocytes were isolated from the whole blood by centrifugation and multiple washing with 0.9% NaCl. PBMCs were isolated from the leukocyte-platelet coat, according to the protocol described before by Kluska et al. [75], after minor modifications. After the separation of the leukocyte-buffy coat from the lymph and red blood cells, the coat was mixed at a ratio of 1:4 with PBS-EDTA (phosphate buffer (0.103 M Na_2_HPO_4_·12H_2_O, 0.154 M NaH_2_PO_4_·H_2_O), 0.9% NaCl and 2 mM sodium-EDTA). Prior to washing the cells in PBS, the cells were additionally treated with erythrocyte lysis buffer (150 mM NH_4_Cl, 10 mM NaHCO_3_, 1 mM sodium-EDTA). Finally, cells were suspended in RPMI-1640 (500 mL of sterile RPMI-1640 with L-glutamine mixed in 5 mL of 1% Pen-Strep and 50 mL FBS) and incubated for 24 h in a culture bottle in 37 °C, CO_2_ 5%vol (MIDI 40 CO_2_ Incubator, Thermo Fisher Scientific, Waltham, MA, USA).

#### 4.2.4. Interaction of the Flavonoids with Human Erythrocytes

##### Hemolytic Activity of the Flavonoids and Their Impact on the Osmotic Resistance of the Erythrocytes

Hemolytic activity and their effect on the osmotic resistance of erythrocytes of the flavonoids were determined according to the procedures described before by Cyboran et al. [76]. Flavonoids were dissolved in DMSO and used in concentrations 50–200 µM and 100 μM, respectively.

##### Changes in the Shape of the Erythrocytes Induced by Flavonoids

To determine the changes of the shapes of the erythrocytes induced by N, Nr and Nd, the experiment was performed according to the procedure described before in Bonarska-Kujawa et al. [77], with minor modifications. Hematocrit was settled at 12%. The concentration of the flavonoids was 100 μM and the control sample was with the addition of DMSO. The blood shapes were observed under an optical microscope Nikon ECLIPSE E200 (Nikon Europe B.V., Amstelveen, The Netherlands) with attached camera, MOTICAM S6 (MoticEurope, S.L.U., Barcelona, Spain). The shapes were distinguished according to the Bessis scale [33] and divided into discocytes, echinocytes and stomatocytes.

##### Effect of Flavonoids on the Trans-Membrane Potential of the Erythrocytes

The determination of changes of the trans-membrane potential, caused by the flavonoids, was performed based on the method described before by Zavodnik et al. [39]. The experiment was performed on fresh erythrocytes. The blood was washed a minimum of 3 times in PBS 310 mOsm buffer. The concentration of the flavonoids used in the experiments were 100 and 200 μM. The fluorescence was measured on a Varian Cary Eclipse Fluorescence Spectrophotometer (Varian, Inc., Palo Alto, CA, USA).

##### Inhibition of Free Radical-Induced Hemolysis by Flavonoids

The experiment testing the ability of the flavonoid to inhibit induced oxidation of the erythrocytes was performed according to the procedure described by An et al. [78], after modifications. The blood was washed a minimum of 3 times in 0.9% NaCl, and the hematocrit was settled at 2.4%. Then, 2 mL of the erythrocytes were incubated with different concentrations of the compounds or standard antioxidants (AA dissolved in phosphate buffer and BHA dissolved in DMSO for 1 h at 37 °C). Meanwhile, 2 mL of the 100 mM solution of AAPH or 400 mM of H_2_O_2_ solution in 0.9% NaCl were prepared. After the incubation, the freshly prepared oxidant solutions were added to the erythrocytes, and the mixtures were incubated for 1 h at 37 °C. Then, 300 µL of the erythrocytes was added to 2.7 mL of the 0.9% NaCl (spekol sample—2.7 mL of water) and centrifuged for 15 min, at 2500 rpm, at 4 °C. The absorbance of the supernatant was measured at 412 nm.

##### Ability of Flavonoids to Prevent the Glutathione Oxidation in Human Erythrocytes

In this experiment, both the ability of the flavonoids to oxidize the glutathione and their protective effect against AAPH-induced glutathione oxidation were checked. Moreover, standard antioxidants AA and BHA, dissolved in DMSO, were checked. The experiment was performed according to Ellman’s procedure [79], after minor modifications. Hematocrit was settled at 10% in NaCl. A total of 2 mL of the cells were incubated with 100 and 200 µM of the flavonoids/antioxidants for 1 h at 37 °C. To the samples used for the second part of the experiment, 60 mM AAPH was added, and they were incubated for 20 min at room temperature. The samples were incubated for 30 min in a dark place at room temperature, and the absorbance was measured at 412 nm.

#### 4.2.5. Effect of Flavonoids on the Mitochondrial Activity of the Human Peripheral Blood Mononuclear Cells (PBMCs) and Human Platelets

After freshly isolated peripheral blood mononuclear cells (PBMCs) were incubated for 24 h, they were transferred into the Falcone and dissolved to a concentration so that 1 × 10^4^ cells were introduced in one hole of a 96-well plate. The compounds were dissolved in RPMI-1640 with Pen-Strep and FBS in order to obtain the final concentration in the hole ranging from 50 to 200 µM in a final volume of 100 µL. The cells were incubated with the compounds for 90 min and 24 h (37 °C, CO_2_ 5% vol). Then, XTT solution with PMS activator was added to each hole. Cells were incubated for 4 h, and the absorbance was measured at 690 nm and 450 nm (blank) on the microplate reader.

The experiments on human platelets were performed using the platelet-rich plasma. The platelets were centrifuged for 10 min, at 1800 rpm, at room temperature. The serum was discarded, and cells were disposed in RPMI-1640 in the volume so that their concentration in the hole was 1 × 10^7^/mL. The concentration of the compounds used in the experiment were 50–200 µM. After the incubation of the cells with the compounds, XTT with PMS was added, and the absorbance at 690 and 450 nm were measured after 24 h.

#### 4.2.6. Interactions of Flavonoids with Human Platelets

##### Ability of Flavonoids to Induce Apoptosis and Necrosis in Platelets

The ability of flavonoids to inhibit apoptosis of human platelets was measured using Annexin/PI staining, according to the procedure described by the Annexin FITC/PI manufacturer. First, platelets were diluted to a concentration around 500 × 10^6^/mL with serum. Before staining, platelets were incubated with the flavonoids in the concentration of 100 μM for 30 min. The concentration of the cells in the sample was 1 × 10^6^/mL. The % of viable, apoptotic and necrotic cells was measured on FACS Calibur flow cytometer (Becton Dickingson, San Jose, CA, USA). The data were processed by the FCS Express v.7 program (DeNovo software, Pasadena, CA, USA).

##### Antiplatelet Activity of Flavonoids

Platelets were centrifuged (3500 rpm for 10 min at room temperature) and diluted with the serum to obtain the number of cells around 500 × 10^6^/mL. Cells were incubated with the compounds for 30 min at 37 °C, mixing. The samples included: PPP (platelet-poor plasma), PRP (platelet-rich plasma). The control cells were placed in aggregation measurement using Chronolog 700. First, 25 mM CaCl_2_ was added to PRP, and the baseline was corrected. Then, 1 mM collagen was added, and the aggregation was measured for 10 min after the addition of collagen. The maximum aggregation of the platelets obtained in the 10 min period was calculated.

#### 4.2.7. Interactions of Flavonoids with Model Lipid Membrane

##### Preparation of Liposomes from the Extracted Lipids from Human Erythrocyte Membranes

Membranes of the erythrocytes were obtained according to the procedure developed by Dodge [80]. Lipids from the human erythrocyte’s membranes were prepared according to the procedure described before by Maddy [81]. The butanol phase was evaporated, and the remaining precipitate was dissolved in a mixture of methanol:chloroform 1:1. The lipids from the isolated erythrocyte membrane lipids were evaporated, hydrated in phosphate buffer and extruded using the pressure extruder Lipex^®^10 mL (Evonik Industries AG, Birmingham, AL, USA) through Whatman™ Nuclepore™ filters (Cytiva, Global Life Sciences Solutions Poland Sp. z o.o., Kraków, Poland) to obtain liposomes with a diameter around 100 nm. The concentration of the liposomes was adjusted to the final 0.2 mg/mL concentration.

##### Effect of Flavonoids on the Hydrodynamic Diameter (HD) and Polydispersity Index (PDI) of the Liposomes

To determine the potential changes in hydrodynamic diameter and polydispersity index (PDI) of the liposomes, induced by the flavonoids, 2 mL of the liposome mixture was incubated for 1 h with 25 and 50 µM of the compounds at room temperature. Then, the parameters were measured using Horiba SZ-100 v2 (Horiba Ltd., Kyoto, Japan).

##### Effect of Flavonoids on Liposomes’ Dipole Potential (ψ_d_)

The effect of the compounds on the dipole potential of the liposomes was tested on the liposome mixture using the fluorescent probe Rh421, according to the procedure described before by Cyboran-Mikołajczyk et al. [70]. Flavonoids were used in the concentrations 25–100 µM.

#### 4.2.8. Statistical Analysis

Statistical analysis was performed using Past 4.16c [82]. Tukey’s test was applied to determine the changes between control and compound-modified samples, with *p* < 0.05.

## 5. Conclusions

N, Nr, and Nd are non-toxic to erythrocytes, peripheral blood mononuclear cells, and platelets. Furthermore, they do not induce apoptosis in platelets and have no effect on the hydrodynamic diameter or polydispersity index of liposomes produced from lipids extracted from erythrocyte membranes. However, the results reveal a strong correlation between their chemical structures and biological activities (structure–activity relationships, SARs). Among the tested compounds, N has the highest lipophilicity but exhibits the lowest antiradical activity. Nd, with moderate lipophilicity, demonstrates the best antiradical activity, while Nr, with the lowest lipophilicity, shows antiradical activity that is better than N’s but lower than Nd’s. N inhibited free radical-induced hemolysis more effectively than Nr and Nd and also increased GSH levels in erythrocytes. However, Nd demonstrated slightly better results in the GSH experiment. Nr showed the strongest antiplatelet activity at the tested concentrations, while Nd inhibited collagen-induced aggregation at lower concentrations than N. Moreover, only N and Nd induced changes in both the trans-membrane potential of erythrocytes and the dipole potential in liposomes. In conclusion, naringenin derivatives exhibit notable biological activity in relation to the tested blood cells. The results obtained for naringin dihydrochalcone are particularly promising, as this flavonoid shows superior protective effects on blood cells compared to N, protecting them against processes that contribute to atherosclerosis. Therefore, Nd could be considered a potential protective compound for blood cells, with potential applications in the development of anti-atherosclerotic therapeutics in the future.

## Figures and Tables

**Table 1:** Partition coefficient values for naringenin (N), naringin (Nr), naringin dihydrochalcone (Nd). The logP values were determined experimentally and obtained from the SwissADME (consensus logP) and MoleInspiration Cheminformatics (milogP) tools. Solubility of N, Nr and Nd was determined using the SwissADME tool (consensus logS). Percentage of DPPH reduction by N, Nr and Nd was in the concentration of 100 μM.

Partition Coefficient				Solubility in Water	DPPH Reduction
Compound	logP_o/w_ ± SD	Consensus logP	milogP	Consensus logS	% ± SD
*N*	1.54 ± 0.21	1.84	2.12	−3.63	9.3 ± 0.1
*Nr*	0.26 ± 0.01	−0.87	−1.58	−2.43	14.1 ± 0.2
*Nd*	0.88 ± 0.09	−0.93	−1.04	−2.53	27.7 ± 0.7

**Table 2:** Percentage of apoptotic and necrotic platelets in the untreated sample (control) and treated with 100 μM of naringenin (N), naringin (Nr) and naringin dihydrochalcone (Nd).

Flavonoid	Apoptotic Cells, % ± SD	Necrotic Cells, % ± SD
*control*	3.9 ± 1.9	1.0 ± 0.7
*N*	1.4 ± 0.1	0.2 ± 0.1
*Nr*	2.6 ± 0.3	0.2 ± 0.1
*Nd*	1.6 ± 0.4	0.2 ± 0.2

## Data Availability

The data presented in this study are openly available in the Base of Knowledge of Wrocław University of Environmental and Life Sciences at https://bazawiedzy.upwr.edu.pl/info/researchdata/UPWR2f3df65889c0471dae02c296e1fad6e2?r=researchdata&ps=20&tab=&title=Dane%2Bbadawcze%2B%25E2%2580%2593%2BDane%2Bbadawcze%2B%25E2%2580%2593%2BUniwersytet%2BPrzyrodniczy%2Bwe%2BWroc%25C5%2582awiu&lang=pl (accessed on 30December 2024).

## Supplementary Materials

The following supporting information can be downloaded at: https://www.mdpi.com/article/10.3390/molecules30030547/s1, Figure S1. Hemolytic curves for control erythrocytes and treated with naringin dihydrochalcone (Nd) in the concentration of 100 uM. *—statistically significant differences between control and Nd-modified erythrocytes with *p* < 0.05. Figure S2. Representative flow cytometry charts obtained for the control platelets (a) and platelets treated with 100 μM of naringenin (b), naringin (c) and naringin dihydrochalcone (d). The percentage of apoptotic cells are presented in the down-right panel (blue) and necrotic cells in the up-right panel (red). Table S1. Percentage of platelet aggregation induced by collagen after treatment with increasing concentrations of naringenin (N), naringin (Nr) and naringin dihydrochalcone (Nd). Table S2. Values of hydrodynamic diameter (HD) in nm and polydispersity index (PDI) of liposomes modified with naringenin (N), naringin (Nr) and naringin dihydrochalcone (Nd) in the concentrations of 25 and 50 μM.
