# Supplementary material for: Insights on the Mechanisms of the Protective Action of Naringenin, Naringin and Naringin Dihydrochalcone on Blood Cells in Terms of Their Potential Anti-Atherosclerotic Activity
Source: Molecules. 2025 Jan 25;30(3):547. doi: 10.3390/molecules30030547 (PMC11820682; doi:10.3390/molecules30030547)
Supplement: Supplementary file 1 [file molecules-30-00547-s001.zip › molecules-3412789-supplementary.pdf]

Supplementary materials

Article title: Insights of the mechanisms of protective action of naringenin, naringin and naringin dihydrochalcone on blood cells in terms of their potential anti-atherosclerotic activity
Journal name: Molecules

Authors: Teresa Kaźmierczak, Sylwia Cyboran-Mikołajczyk, Natalia Trochanowska-Pauk, Tomasz Walski, Paulina Nowicka, Dorota Bonarska-Kujawa
Affiliation and e-mail address of the corresponding author: Department of Physics and Biophysics Faculty of Biotechnology and Food Sciences, Wrocław University of Environmental and Life Science, Norwida 25 St., 50-375 Wrocław
Correspondence: dorota.bonarska-kujawa@upwr.edu.pl

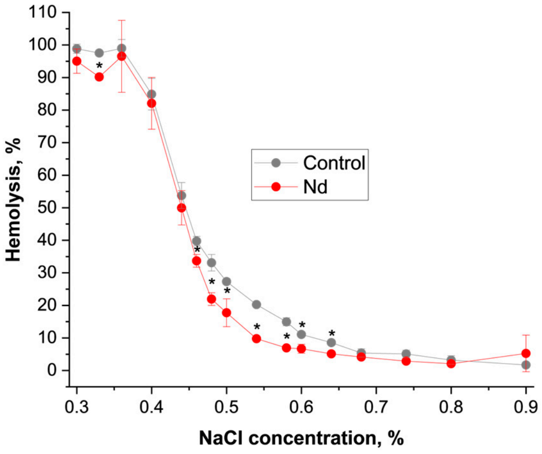

Figure S1. Hemolytic curves for control erythrocytes and treated with naringin dihydrochalcone (Nd) in the concentration of 100 uM. *—statistically significant differences between control and Nd-modified erythrocytes with p<0.05.

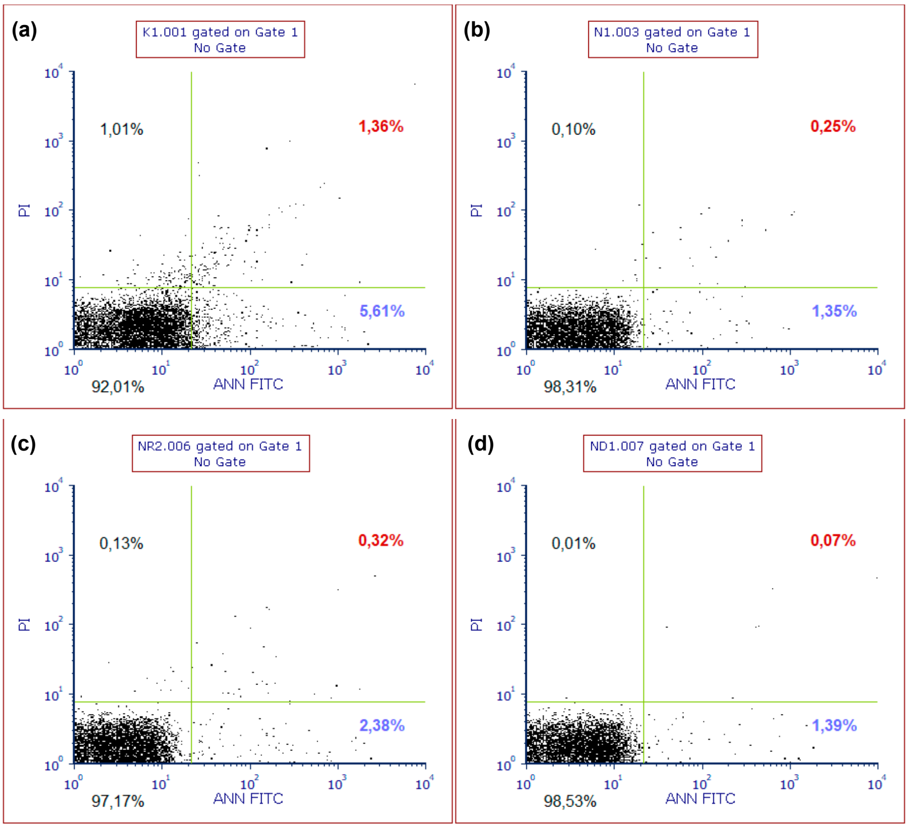

Figure S2. Representative flow cytometry charts obtained for the control platelets (**a**) and platelets treated with 100 µM of naringenin (**b**), naringin (**c**) and naringin dihydrochalcone (**d**). The percentage of apoptotic cells are presented in down-right panel (blue) and necrotic cells in up-right panel (red).

Table S1. Percentage of platelet aggregation induced by collagen after the treatment with increasing concentrations of naringenin (N), naringin (Nr) and naringin dihydrochalcone (Nd).

**Antiplatelet activity**	
**Concentration, µM**	**Aggregation, % ± SD**
N	
25	81.0± 10.1
50	15.7 ± 6.1
100	0.7 ± 0.1
Nr	
25	0.63 ± 0.05
50	0.82 ± 0.18
75	0.50 ± 0.44
125	0.56 ± 0.47
Nd	
25	84.1 ± 14.8
50	36.0± 7.0
75	0.3 ± 0.2
100	0.6 ± 0.2

Table S2. Values of hydrodynamic diameter (HD) in nm and polidyspersity index (PDI) of liposomes modified with naringenin (N), naringin (Nr) and naringin dihydrochalcone (Nd) in the concentration of 25 and 50 μM.

**Compound, 25 μM**	**HD, nm ± SD**	**PDI, ± SD**
control	113.3 ± 2.6	0.16 ± 0.02
Nr	113.8 ± 4.9	0.13 ± 0.04
Nr	116.6 ± 2.8	0.18 ± 0.02
Nd	117.2 ± 3.4	0.20 ± 0.02
**Compound, 50 μM**	**HD, nm ± SD**	**PDI, ± SD**
control	113.3 ± 2.6	0.16 ± 0.02
N	119.6 ± 2.1	0.22 ± 0.05
Nr	116.8 ± 3.3	0.15 ± 0.02
Nd	115.9 ± 4.0	0.18 ± 0.02
